# The Accuracy and Cost-Effectiveness of MRI Assessment of Collateral Ligament Injuries of the Lesser Digits’ Proximal Interphalangeal Joints

**DOI:** 10.7759/cureus.28306

**Published:** 2022-08-23

**Authors:** Mehmet S Sahin

**Affiliations:** 1 Orthopedics and Traumatology, Baskent University Alanya Research and Practice Center, Antalya, TUR

**Keywords:** diagnostic studies, proximal interphalangeal joint injury, finger injury, mri imaging, lesser digit collateral ligament injury

## Abstract

Background

Collateral ligament injuries of the thumb and lesser digits are simple injuries, but they may lead to disabilities in hand function. This study aimed to evaluate the accuracy and cost-effectiveness of magnetic resonance imaging (MRI) in diagnosing proximal interphalangeal (PIP) collateral ligament injuries of lesser digits.

Methods

A retrospective evaluation was conducted on 18 fingers that had undergone surgery for PIP joint complete collateral ligament injury. Pre-operative MRI results were compared with the intra-operative findings. The data from MRI and direct intraoperative findings were analyzed by the Chi-square test in paired groups. The McNemar test analyzed the accuracy of the MRI test for detecting volar plate injuries. Statistical Packages for Social Sciences (SPSS) version 25 (IBM Inc., Armonk, New York) software program was used for the analysis.

Results

In digits other than the thumb, the accuracy of MRI for detecting collateral injuries was 38.89%, and detection was incorrect in 11 (61.11%) of 18 patients. There are significant differences between MRI and Intraoperative results (p<0.001). MRI findings for seven fingers (38.89%) of the 18 fingers involved were compatible with the surgery results (38.88%). By comparison, the MRI findings of 11 fingers (61.11%) were inconsistent with the intra-operative results. Eight patients (44.44%) were diagnosed preoperatively with MRI as having volar plate ruptures, three patients (16.67%) were diagnosed with open surgery, but only three of the volar plate diagnosed patients with MRI were verified as ruptures during open surgery (38.0%). In addition, preoperatively undetected volar plate injuries by MRI (n=10) were detected intra-operatively in three cases (30.0%). Therefore, the accuracy of MRI was found not to be statistically significant for the detection of volar plate injuries (p=0.727).

Conclusion

This study concluded that a 1.5-Tesla MRI with a slice thickness of 2-3 mm should not be relied on as a decisive tool for diagnosing collateral ligament injuries of the PIP joint of the lesser digits. Additionally, MRI was found insufficient for diagnosing volar plate injuries that accompanied collateral ligament injuries. Given these findings, one might conclude that MRI is not cost-effective in diagnosing collateral ligament injuries of the lesser digits PIP joint.

## Introduction

Collateral ligament injuries of the thumb and lesser digits are simple injuries, but they may lead to disabilities in hand function [1]. Unfortunately, inexperienced surgeons can easily overlook this condition. Although the diagnosis is still clinical, magnetic resonance imaging (MRI) is the preferred imaging method [1]. MRI has shown high accuracy in evaluating thumb metacarpophalangeal (MCP) joint collateral ligament injuries [2]. The collateral ligament tear rates of the lesser digits were lower than those of the collateral ligament of the thumb [3]. There is no consensus on the criteria to establish whether the collateral ligament laxity seen in the lesser digits is pathological or within normal limits.

Therefore, surgical indications for this type of injury remain a matter of debate among scientists in the literature. The laxity of the thumb collateral that necessitates repair can be considered a normal laxity in the lesser digits [4]. When deciding on treatment, it is critical to recognize a partial or complete rupture [5]. Partial rupture can be treated conservatively, whereas complete ligament ruptures are treated surgically [6]. The ability of MRI to distinguish between partial and complete ruptures may aid clinical decision-making regarding treatment. This study aimed to evaluate the accuracy of MRI in the diagnosis of collateral ligament injuries to lesser digits.

## Materials and methods

A retrospective evaluation was conducted on 18 fingers that had undergone surgery for proximal interphalangeal joint complete collateral ligament injury. The study was approved by the Ethics Committee of the Baskent University Institutional Review Board and registered under the protocol. Informed consent was obtained from all the patients before their enrollment in the study.

In this study, the list of operated patients (for proximal interphalangeal collateral ligament injuries of lesser digits) was searched from the digital archive of the hospital. Those who had undergone a preoperative MRI scan were separated from this list and included in the study. Those who were diagnosed only by physical examination and stress radiographs but did not have an MRI were excluded from the study.

Between 2012 and 2020, the cases of 18 patients, 11 men and seven women, were evaluated retrospectively. Two cases with late admission (≥90 days) were determined as chronic cases [7,8].

The clinical decision regarding the presence of a collateral ligament rupture of the PIP joint of the lesser digits was made with the joint extended with flexion of 30°, as described by Minamikawa et al. [9]. This study found that a lateral angulation of 10° in extension and/or 20° of lateral angulation at 30°of flexion indicated complete collateral ligament rupture. In addition, the absence of an endpoint during stress loading was a contributing factor.

Inclusion and exclusion criteria

The study included injuries that were diagnosed through physical examination and MRI as collateral ligament total rupture of the PIP joint. The study excluded injuries in the MCP joint of all fingers, injuries in the collateral ligament of the proximal interphalangeal (PIP) joint associated with bony fragment and open injuries, or fingers that did not fulfill Minamikawa et al.’s criteria for complete rupture [9].

The MRI examination costs were granted by this study’s university.

Preoperative MRI results were compared with the direct intra-operative findings. The data obtained from MRI and direct intra-operative findings was analyzed through the Statistical Packages for Social Sciences (SPSS) version 25 (IBM Inc., Armonk, New York) software program, McNemar’s test, and Chi-square test. The diagnostic accuracy of chronic and subacute/acute cases with the MRI was analyzed through Fisher’s exact test.

**Figure 1:**
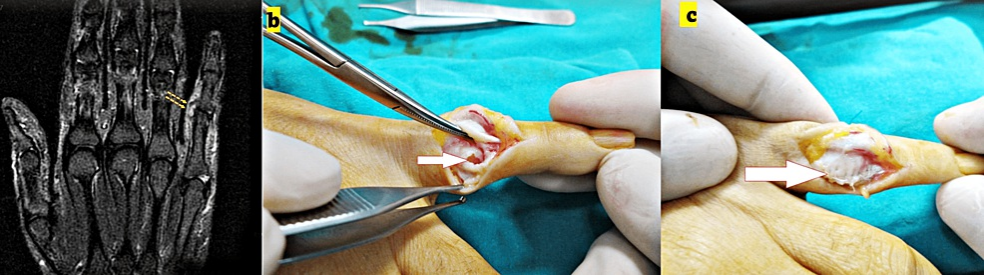
Preoperative MRI and intra-operative photograph of fingers (a) Preoperative MRI image, a coronal T2-weighted image showing the radial collateral tear of the fifth finger (yellow arrows) located in the joint proximal interphalangeal (PIP). The MRI images were reported as total rupture and verified during surgery. (b,c) Intra-operative photographs showing the repaired ligament.

**Figure 2:**
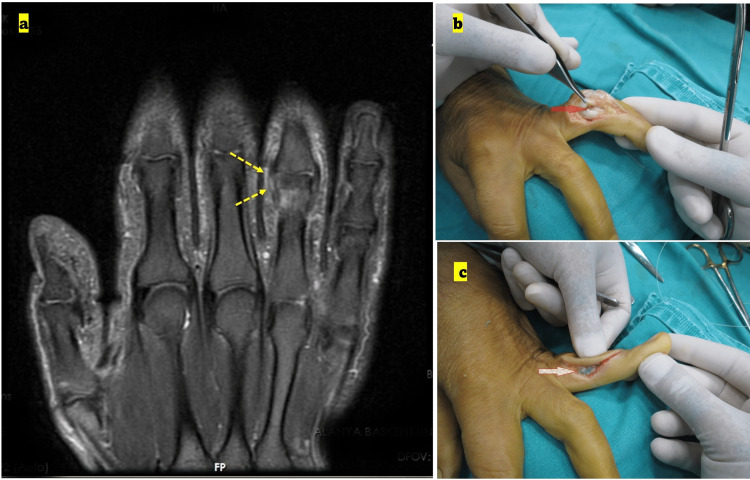
Preoperative MRI and intra-operative photograph of fingers (a) Preoperative MRI image, a coronal T2-weighted image showing the radial collateral injury of the fourth finger (yellow arrows) at the proximal interphalangeal (PIP) joint. The MRI images were indicated as partial rupture and were not verified during surgery. (b) Intra-operative photograph showing total rupture (no correlation with the preoperative MRI findings. (c) Intra-operative photograph showing the repaired ligament.

**Figure 3:**
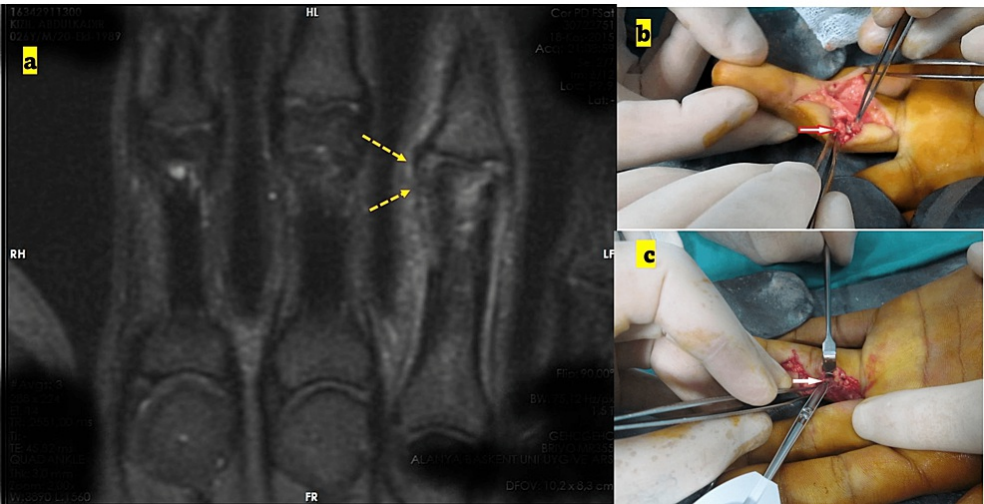
Preoperative MRI and intra-operative photograph of fingers (a) Preoperative MRI image, a coronal T2-weighted image showing the radial collateral injury of the fourth finger (yellow arrows) in the joint proximal interphalangeal (PIP). The MRI images were indicated as partial rupture and were not verified during surgery. (b) Intra-operative photograph showing total rupture collateral ligament with volar plate avulsion (no correlation with the preoperative MRI findings). (c) Intra-operative photograph showing the timing of the use of the suture anchor for repair.

Preoperative MRI evaluations and data regarding the degree of collateral ligament rupture were obtained from the report of a senior radiologist (Figures 1, 2, 3).

The data regarding existing complete or partial ligament injuries was verified or unverified by the intra-operative examination (Figures 1, 2, 3). MRI examination of the hand was performed with a 1.5-Tesla scanner, with a 100 mm wrist coil and slice thickness of 2-3 mm (Inspire MR 355; GE Healthcare, Chicago, Illinois; produced on June 12, 2013).

The imaging methodology and its parameters included a three-level scout: sagittal T1-weighted images (T1WI, T2WI), axial (T1WI and T2WI), coronal (T1WI, T2WI, proton density with fat saturation and short TI inversion recovery (STIR)).

Acute collateral ligament tears are diagnosed using MRI criteria that include ligament discontinuity, detachment, or thickening, as well as increased intra-ligamentous signal intensity on T2-weighted images, which might indicate edema or bleeding. The fat planes around the ligament may be obliterated, and synovial fluid may flow into the neighboring soft tissues. Chronic tears frequently result in ligament thickening, which is related to scarring. The ligament may also show signs of thinning, strain, or an undulating shape [10-12].

Acute volar plate tears are diagnosed using MRI criteria that include nonhomogeneous signal intensity on T1 and T2-weighted images, together with thickening and contour irregularities. Also, detecting disruption volar plate attachment with a gap was another criterion of MRI diagnosing [10].

Patients were supine on the MRI table, and the position of the hand was fixed in a neutral position with pads. The patients were operated on by a single surgeon using the same approach in each case. The dorsolateral approach to the PIP joint was used for lesser digits, and a wide-based “V” shape incision was made. The skin was retracted dorsally as a full-thickness flap (Figures 1, 2, 3).

The extensor mechanism and capsule were incised, and the ruptured collateral ligament was repaired (Figures 1, 2, 3). Collateral ligament injuries were observed in almost all cases, and the location of the rupture was at the origin or insertion points of the ligaments. A mini corkscrew (suture anchor) was used for fixation (1.3 De-Puy Mitek; Johnson & Johnson, New Brunswick, New Jersey; Figure 3). The extensor mechanism and capsule were repaired, and the skin was closed with mattress sutures.

In a case of a late admission (150 days) in which there was a tear of the radial collateral ligament (PIP-RCL) of the fifth finger, an adjustment was made in the reconstruction of the oblique retinacular ligament, which was consistent with the study of Littler and Colley [13].

Following surgery, the joint was immobilized with a splint for two weeks, and the patient was later referred to a hand physiotherapist. Informed consent was obtained from all the patients before their enrollment in the study.

## Results

The mean age was 52.4 ±17.8 years for females and 37.45±11.5 for males. The dominant and non-dominant ratios were equal in both groups. The injury types and locations detected in the collateral ligament on MRI are shown in Table 1. 

**Table 1:** Demographic data, preoperative MRI, and intra-operative findings of patients who underwent repair of the lesser digit collateral ligament RCL - radial collateral ligament, UCL - ulnar collateral ligament, PIP - proximal interphalangeal

Patient	Age	Gender	Preoperative duration (day)	Side	Injured finger	Injured ligament	Preoperative MRI evaluation	Intraoperative correlation
1	62	f	60	Left	4th finger PIP	RCL	RCL partial rupture, volar plate injury	No (RCL has been observed as total rupture and volar plate injury has not been verified)
2	31	m	10	Right	3rd finger PIP	UCL + volar plate	UCL partial rupture	No (UCL rupture has been verified total and additionally volar plate rupture detected)
3	29	f	3	Left	5th finger PIP	RCL	Volar plate injury and RCL partial rupture	No (volar plate injury has not been verified and RCL has been observed as total rupture)
4	49	f	45	Right	5th finger PIP	RCL + volar plate	Volar plate injury and RCL total rupture	Yes (volar plate and RCL total rupture have been verified)
5	33	m	150	Left	5th finger PIP	RCL	Volar plate injury and RCL partial rupture	No (volar plate injury has not been verified and RCL has been observed as total rupture)
6	65	f	90	Left	4th finger PIP	RCL	RCL partial rupture	No (RCL has been observed as total rupture)
7	32	m	30	Right	4th finger PIP	UCL	UCL total rupture	Yes (UCL has been observed as total rupture)
8	41	m	42	Left	3rd finger PIP	UCL	Volar plate injury and UCL partial rupture	No (volar plate injury has not been verified and UCL has been observed as total rupture)
9	51	m	15	Right	5th finger PIP	RCL + volar plate	Volar plate injury and RCL total rupture	Yes (volar plate and RCL total rupture have been verified)
10	27	m	10	Left	3rd finger PIP	UCL + volar plate	UCL partial rupture	No (volar plate injury has been observed and UCL has been observed as total rupture)
11	26	m	4	Left	4th finger PIP	RCL + volar plate	Volar plate injury and RCL total rupture	Yes (volar plate and RCL total rupture have been verified)
12	27	m	5	Left	2nd finger PIP	UCL + volar plate	UCL partial rupture	No (UCL has been observed as total rupture and volar plate has been observed as total rupture)
13	61	f	30	Left	5th finger PIP	RCL	RCL total rupture	Yes
14	28	f	30	Left	4th finger PIP	UCL	Volar plate injury and UCL partial rupture	No (volar plate injury has not been verified and UCL has been observed as total rupture)
15	53	m	7	Left	4th finger PIP	RCL	RCL total rupture	Yes
16	73	f	12	Right	3rd finger PIP	RCL	RCL total rupture	Yes
17	58	m	42	Right	2nd finger PIP	RCL	No ligament rupture detected	No (RCL has been observed as total rupture)
18	33	m	28	Left	3rd finger PIP	UCL	UCL partial rupture	No (UCL has been observed as total rupture)

The distribution of patients in terms of age, sex, affected sides, and pre-op duration of symptoms (days) are shown in Table 2.

**Table 2:** The distribution of patients according to characteristics

		Female n=7 (38.9%)	Male n=11 (61.1%)	Total N=18
Age (p=0.085)	Mean	52.43	37.45	43.28
	Std. deviation	17.81	11.49	15.69
	Median	61	33	37
	Minimum	28	26	26
	Maximum	73	58	73
Preoperative duration (day) (p=0.328)	Mean	38,57	31,18	34,06
	Std. deviation	29,62	41,87	36,81
	Median	30	15	29
	Minimum	3	4	3
	Maximum	90	150	150
		n (%)	n (%)	n (%)
Side (p=0.999)	Left	5 (71.4)	7 (63.6)	12 (66.7)
	Right	2 (28.6)	4 (36.4)	6 (33.3)
	Total	7 (100.0)	11 (100.0)	18 (100.0)

The imaging methodology and its parameters are shown in Table 3. 

**Table 3:** MR imaging parameters were used in the study TR - repetition time, TE - echo time, FOV - field of view, STIR - short tau inversion recovery, PD - proton density, msec - millisecond, sec - second, mm - millimeter, cm - centimeter

Pulse sequences	T1WI	T2WI	STIR	PD
TR	520 ms	3200 ms	3000 ms	2900 ms
TE	13 ms	84 ms	47 s	46 ms
Slice thickness	3mm	3 mm	2 mm	2.5 mm
Interslice gap	2-3 mm	2-3 mm	2-3 mm	2-3 mm
Matrix size	288-224 pixels	288-224 pixels	288-224 pixels	288-224 pixels
FOV	16 cm	16 cm	16 cm	16 cm
Time of examination	2-3 min	2-3 min	5 min	5 min

The accuracy of MRI as a diagnostic tool for diagnosing ulnar collateral ligament damage was found to be more than 28.6%, while detecting radial collateral injuries with an accuracy of 70% (Tables 1, 4). The distribution of the locations of injured fingers is presented in Table 4, and the locations of injuries that MRI had not diagnosed are shown in Tables 1 and 4.

**Table 4:** Preoperative and intraoperative findings RCL - radial collateral ligament, UCL - ulnar collateral ligament

Injured finger	Preoperative MR evaluation	n	Intra-operative evaluation	Concordance
Second finger (n=2)	RCL partial rupture	1	RCL total rupture	-
	UCL partial rupture	1	UCL total rupture + volar plate injury was detected	-/+
Third finger (n=5)	RCL total rupture	1	RCL total rupture	+
	UCL partial rupture	1	UCL total rupture	-
	UCL partial rupture	1	UCL total rupture + volar plate injury was detected	-/+
	UCL partial rupture	1	UCL total rupture + volar plate injury was detected	-/+
	UCL partial rupture + volar plate injury	1	UCL total rupture + volar plate injury not verified	-/-
Fourth finger (n=6)	RCL total rupture	1	RCL total rupture	+
	RCL partial rupture	1	RCL total rupture	-
	UCL total rupture	1	UCL total rupture	+
	RCL partial rupture + volar plate injury	1	RCL total + volar plate injury not verified	-/-
	RCL total rupture+ volar plate injury	1	RCL total rupture + volar plate injury	+/+
	UCL partial rupture + volar plate injury	1	UCL total ruptue+ Volar plate injury not verified	-/-
Fifth finger (n=5)	RCL total rupture	1	RCL total rupture	+
	RCL partial rupture + volar plate injury	1	RCL total rupture + volar plate injury not verified	-/-
	RCL Total rupture + volar plate injury	1	RCL total rupture + volar plate injury verified	+/+
	RCL partial rupture + volar plate injury	1	RCL total rupture + volar plate injury not verified	-/-
	RCL Total rupture + volar plate injury	1	RCL total rupture + volar plate injury verified	+/+

Among the patients in the study, total rupture was detected the most in the fifth and fourth digits. Third and second finger injuries were most frequently overlooked on MRI.

In digits other than the thumb, the sensitivity of MRI for detecting collateral injuries was 55.3%, and the positive predictive value was 52.9%. Detection was incorrect in 11 (61.1%) of 18 patients.

In general, MRI findings for seven fingers (38.89%) of the 18 fingers involved were compatible with the results of the surgery (38.88%). In comparison, the MRI findings of 11 fingers (61.11%) were inconsistent with the intra-operative results (Table 4).

Only two cases from our group can be defined as chronic, and the authors of this study investigated the difference between chronic and acute-subacute cases in terms of the diagnostic accuracy of MRI. Fisher’s exact test is used due to the cells with less than five expected values. Since the test statistic result is 0.497 (p>0.05), the null hypothesis that duration has no effect on diagnostic accuracy is accepted.

Eight patients (44.44%) were diagnosed preoperatively with MRI as having volar plate ruptures, three patients (16.67%) were diagnosed with open surgery, but only three of the volar plate diagnosed patients with MRI were verified as ruptures during open surgery (38.0%). In addition, preoperatively undetected volar plate injuries by MRI (n=10) were detected intra-operatively in three cases (30.0%). The accuracy of MRI was found to be not statistically significant for the detection of volar plate injuries (p=0.727). Therefore, the accuracy of MRI was found to be inefficient for the detection of volar plate injuries (Table 4).

The detection of volar plate injuries using MRI is slightly more complicated than the procedure used for collateral ligaments. In addition, volar plate injuries often impair the detection of collateral ligament injuries because of their superimposing effect (Figure 3).

## Discussion

Based on these findings, this study believes that MRI is not a valuable tool for detecting collateral ligament injuries of the PIP joint in lesser digits.

Ligament injuries of MCP joints of lesser digits have been less frequently reported, and studies have shown the unsuccess of MRI in demonstrating lesser digits MCP joint collateral ligament injuries [2]. However, MRI and ultrasound (USG) accuracy was found to be relatively high in the evaluation of thumb MCP pathologies [14-17]. In contrast to the many positive studies that have reported the accuracy of MRI in detecting thumb MCP joint collateral ligament injuries, there are very few publications on the characteristics of MRI for PIP joints of the lesser digits. However, these studies do not focus on the accuracy of MRI in PIP joint collateral ligament injuries [10, 12, 18-21]. Tuncay and Ege reported case series regarding clinical results of suture anchor repair of eight patients, six with the chronic instability of the collateral ligament of the thumb and two with the instability of the fifth finger, using the Statak® suture anchor (Zimmer Biomet, Warsaw, Indiana). The clinical decision regarding the presence of a collateral ligament rupture of the PIP joint of the lesser digits was made with physical examination in their study [22].

Owing to the small size of the fingers and their extraordinarily complex structures, it is not easy to evaluate them through imaging [23]. Radiographs are often negative when there is no fracture, and in these cases, USG and MRI are of increasing diagnostic value. Injury is best appreciated through coronal fluid-sensitive MRI [24]. As with other ligamentous injuries, MRI and USG will demonstrate ligamentous thickening or attenuation with partial-thickness tears and a fluid gap or absence of the ligament with a complete tear [25]. Advances in the quality of MRI and USG techniques have provided a better understanding of the normal and pathological features of the fingers [23, 24, 26, 27]. Hauser et al. and Rozmaryn et al. emphasized that the normal recess at the base of the dorsal capsule may be mistaken for a tear [18, 19]. While MRI may be useful for detecting torn ligaments, it cannot identify ligaments that are loose or have extended more than twice their normal length [19, 27]. It has been stated that MRI is not a powerful device as a diagnostic tool [19, 28].

Ligament thickening and fibrosis may be observed in chronic cases. This has led others to suggest that where an MRI examination is normal, high clinical suspicion and a professional evaluation would be more accurate. In addition, affordable testing in clinics would allow care to continue quickly and most economically without the regular use of MRI [19, 29]. This study suggests that MRI may not accurately diagnose collateral injuries, especially at the PIP joints. 

Few studies have evaluated MCP joint and PIP joint collateral ligament tears of the lesser digits. Gong et al. reported six cases of radial collateral ligament tears in the PIP joint of the fifth finger due to repetitive stress in young pianists [20]. They performed a physical examination for diagnosis rather than MRI [20]. Kang et al. reported the results of 12 patients who underwent surgery for index finger MCP joint radial collateral ligament tears. They found that the findings obtained before surgery were similar to those observed during surgery [30].

A study by Theumann et al. reported that MRI, using a 1.5 T unit (Signa; GE Healthcare, Chicago, Illinois) with a phased-array wrist coil or a home-built local gradient coil, accurately depicted not only injuries of the collateral ligaments but also associated lesions of MCP joint injuries [31]. Indeed, the authors emphasized that MRI is a successful tool for detecting injuries [31].

Pfirmann et al. conducted research to determine the role of MR imaging in assessing collateral ligaments of MCP joints of fingers. It was discovered that standard MRI is effective for viewing these structures. In addition, the author found that MRI was useful for identifying damage to collateral ligaments of MCP joints [32]. Studies with three tesla MRI have emerged recently; these studies have been more of a descriptive nature. Gupta et al. published an article in 2015 that reviewed the MRI anatomy of the fingers and discussed common tendon and ligament injuries [33].

Lewis et al. [34] performed a study to correlate MRI of the PIP joint with its anatomy. They described anatomic structures and their reflections on MRI. However, their focus was to define the morphologic features of the cross-sectional view of the PIP joint and its reflections on MRI. Petchprapa et al., Clavero et al., and Abdellatif et al. studied the typical characteristics of finger MRI and discussed the problems associated with MRI for finger injuries [10,12,21].

The literature has many articles that focus on the accuracy of MRI for thumb MCP joint ligament injuries. However, there is no work in the current literature regarding the accuracy of MRI in detecting ligamentous injuries of the PIP joint. Thus, this study contributes to the literature, especially regarding the accuracy of MRI in PIP joint ligament injuries.

Regarding the diagnostic value of physical examination and stress radiography, there are two different views in the literature. Some authors, contrary to the general opinion, reported weak evidence [35], while the majority of publications emphasized the importance of the classical methods.

The majority of ligamentous lesions in the MCP joint of the thumb can be discovered indirectly using physical stress testing and stress radiography, which are considered first-line investigative techniques when the injuries cause gross instability [1]. Stress radiography can be used to determine whether a complete ligamentous tear is present. However, there is considerable disagreement not only regarding its ideal performance but also regarding the determination of the location of the tear and the diagnosis of associated injuries [36-38].

Besides the differences in performance and interpretation of stress radiographs, their major limitation is that they do not lead in the treatment of UCL injuries because they cannot differentiate between a nondisplaced rupture and a Stener lesion [36,39]. Nevertheless, when the instability is subtle, additional imaging studies (such as MRI) that can precisely highlight the soft tissue structures may be necessary [40]. In addition to this, MRI can also play a significant part in the surgical planning process, which ultimately results in a better prognosis [36]. 

Even though there is a significant amount of research available about magnetic resonance imaging (MRI), it is still difficult to determine the injury pattern and make an accurate diagnosis in patients who have sustained hand trauma using MRI [15,41,42]. The current study also emphasized that the accuracy rate of MRI is not enough for the diagnosis of PIP joint collateral ligament injuries of lesser digits.

In the literature, Lutsky et al. [2] reported that the specificity of MRI in detecting MCP joint collateral ligament injuries of the lesser digits was 64%, and the positive predictive value was 100%. In contrast, this study examined the collateral ligaments of the lesser digits at the PIP joint and found that the sensitivity of MRI for detecting collateral ligament injuries was 55.3%. Furthermore, the positive predictive value was 52.9%.

Pre-operative MRI findings of 10 fingers in 18 patients were inaccurate compared with intra-operative findings (55.5%). This confirms that MRI is insufficient for detecting injuries to the lesser digits in the PIP joint collateral ligament. The lower accuracy of MRI in diagnosing collateral ligament injuries in lesser digits may be attributed to the small size and complex anatomy of the joint, existing edema, and associated injuries, which might overlap with existing collateral ligament injuries. In addition, scar tissues that occur in chronic injuries may cause superposition of the torn ligament, causing the total tear to be hidden. 

Limitations

The limitations of this study are the retrospective nature, limited number of subjects, and the lack of a control group to compare the results.

A second group could be formed in which partial tears are diagnosed and treated conservatively through physical examination and stress radiography, and patients in this group could undergo MRI, funded by the university's scientific fund, to determine the accuracy with which MRI detects partial tears in this group. The model may be utilized to construct an effective study plan for future research.

## Conclusions

Based on these results, this study concludes that a 1.5 Tesla MRI with a slice thickness of 2-3 mm is not a decisive tool for diagnosing collateral ligament injuries of the PIP joint of the lesser digits. In addition, MRI was found to be inadequate for diagnosing volar plate injuries associated with collateral ligament injuries. Given these results, decision-makers should consider that MRI is not cost-effective in diagnosing collateral ligament injuries of the lesser digits of the PIP joint at this accuracy rate.

The anamnesis and physical examination may be more helpful and cost-effective than an MRI in identifying collateral ligament rupture in the PIP joint of the lesser digits. However, to understand concomitant lesions and to plan appropriately preoperatively, it may be more useful to combine both physical examination and history and additional imaging techniques.
